# Diseases Admitted under the Care of the Physicians of the Westminster Hospital, from the 25th of March to the 27th of April, 1801

**Published:** 1801-05

**Authors:** 


*Difeafes admitted under the care of the Phyficians of the Weftminfter Hofpital, from the 25th of March to the 27th of April,* 1801.

| | No. of Cafes. | | No. of Cafes |
|---|---|---|---|
| Continued Fever - - - - | 8 | Hæmorrhois - - - - - - | 1 |
| Intermittent - - - - - | 1 | Impetigo - - - - - - | 3 |
| Otitis - - - - - - - - | 1 | Ifchuria - - - - - - - | 1 |
| Pleuritis - - - - - - | 2 | Leucorrhœa - - - - - - | 2 |
| Cynanche - - - - - - | 1 | Menorrhagia - - - - - | 1 |
| Anafarca - - - - - - | 4 | Obftipatio - - - - - - | 1 |
| Afcites - - - - - - - | 1 | Paralyfis - - - - - - - | 2 |
| Afthenia - - - - - - - | 3 | Phthifis - - - - - - | 2 |
| Afthma - - - - - - - - | 1 | Prolapfus - - - - - - | 1 |
| Catarrh - - - - - - | 3 | Rheumatifm - - - - - - | 11 |
| Chlorofis - - - - - - - | 1 | Struma - - - - - - - | 3 |
| Diarrhœa - - - - - - | 2 | Syphilis - - - - - - - | 1 |
| Dyfpepfia - - - - | 2 | Tuffis - - - - - - - | 8 |
| Dyfpnœa - - - - - - | 1 | Urticaria - - - - - - | 1 |
| Enterodynia - - - - | 1 | Vermes - - - - - - - | 2 |
| Gaftrodynia - - - - - - | 2 | Vertigo - - - - - - - | 1 |
| Hæmoptœ - - - - - - | 3 | | |

